# Antibacterial activity of *Myrtus communis* leaf extract against three pathogenic *Xylella fastidiosa* subspecies

**DOI:** 10.3389/fpls.2025.1701374

**Published:** 2025-11-20

**Authors:** Giuseppe Incampo, Marwa Mourou, Davide Cornacchia, Vito Montilon, Graziana Difonzo, Paola Montoro, Francesco Caponio, Francesco Faretra, Franco Nigro, Stefania Pollastro

**Affiliations:** 1 Department of Soil, Plant and Food Sciences University of Bari Aldo Moro, Bari, Italy; 2 Department of Pharmacy, University of Salerno, Fisciano, Italy

**Keywords:** *Xylella*-subspecies, biocontrol, qPCR, plant-extract, *Nicotiana benthamiana*

## Abstract

The limited availability of sustainable strategies for managing *Xylella fastidiosa* (Xf) underscores the urgent need for innovative and practical antimicrobial solutions. In this study, an extract from the leaves of *Myrtus communis* (MC) biotype “Tarantino”, known for its antibacterial properties, was evaluated *in vitro* using well diffusion and broth dilution assays against *X. fastidiosa* subsp. *pauca* (Xfp), *multiplex* (Xfm), and *fastidiosa* (Xff), all of which have been reported in Europe and in the Apulia region. Prior to biological testing, the MC leaf extract (MCLE) underwent chemical characterization via LC-Q-Exactive-Orbitrap/MS and LC-Q-Exactive-Orbitrap/MS/MS. Metabolite profiling revealed the presence of several phenolic acids, flavonol derivatives, and ellagitannins. In the *in vitro* assays, MCLE exhibited clear inhibition zones against all three Xf subspecies, with diameters ranging from 4.8 to 22.6 mm compared to the control. Additionally, in broth cultures, a 1:5 dilution of MCLE significantly inhibited bacterial growth, resulting in 65%, 86%, and 66% inhibition for Xfp, Xfm, and Xff, respectively. For *in planta* assays, symptom severity on the canopy of *Nicotiana benthamiana* was notably reduced in plants treated with MCLE compared to those inoculated with Xf alone. Quantitative real-time PCR confirmed the efficacy of MCLE: untreated, inoculated plants exhibited significantly lower Cq values (F = 120; p < 0.001), indicating higher bacterial loads compared to MCLE-treated plants. Overall, this study highlights the potential of MC-based formulations as promising, eco-friendly tools for the management of Xf-related diseases, meriting further validation under field conditions.

## 1 Introduction

*Xylella fastidiosa* (Xf) is a Gram-negative bacterium that consistently causes severe damage to a wide range of economically important crops worldwide, including coffee, citrus, grapevine, olive, and almond. Since its first detection in the Apulia region of Italy in 2013, *X. fastidiosa* subsp. *pauca* (Xfp) has become the most serious threat to olive cultivation in Europe (Velasco-Amo et al., 2023). Xfp infections have led to a dramatic decline in olive yields and tree viability, affecting over 5 million olive trees and resulting in an estimated 10% reduction in Italy’s overall olive oil production (White et al., 2020). The Xfp De Donno strain, associated with Olive Quick Decline Syndrome (OQDS), was isolated in 2013, and its whole genome was subsequently sequenced (Saponari et al., 2013; Giampetruzzi et al., 2015). In addition to Xfp, other *X. fastidiosa* subspecies have recently emerged in Europe. Notably, *X. fastidiosa* subsp. *multiplex* (Xfm) sequence type 26 (ST26) was reported for the first time in spring 2024 on asymptomatic almond trees in southern Italy (Montilon et al., 2024). More recently, *X. fastidiosa* subsp. *fastidiosa* (Xff) ST1 was also detected for the first time in Apulia, Southern Italy (Cornara et al., 2025). The primary pathogenic mechanism of Xf involves the formation of biofilms within the xylem vessels, which disrupts water transport and compromises plant health (Roper et al., 2019; Anbumani et al., 2021). Within the biofilm matrix, bacterial cells coordinate their behavior through quorum sensing, allowing them to adapt, persist, and resist hostile conditions. Current control methods—such as the use of N-acetyl-L-cysteine (NAC), menadione, and copper(II) sulfate—aim to limit the spread and activity of Xf (Mourou et al., 2025; Ge et al., 2020). However, these treatments often provide inconsistent results and are considered unsustainable for long-term management (Scortichini et al., 2021). This highlights the urgent need for novel and environmentally sustainable biocontrol strategies that align with European ecological and regulatory priorities.

Plant-derived compounds represent a promising source of natural antimicrobial agents. Extracts and essential oils from various plant species have shown effective activity against numerous bacterial and fungal pathogens (Rocha et al., 2005; Ayaz et al., 2016). Among these, *Myrtus communis* L. (MC), commonly known as myrtle, is a Mediterranean aromatic evergreen shrub belonging to the Myrtaceae family. Interestingly, MC has been listed among host plants for *Xylella fastidiosa* subspecies *pauca*, *multiplex*, and *fastidiosa* (European Food Safety Authority (EFSA) et al., 2025). However, the evidence of natural infection remains limited and may rely on detection data rather than confirmed pathogenic interaction. Importantly, MC exhibits a broad spectrum of biological activities, including antibacterial, anti-inflammatory, disinfectant, antioxidant, hypoglycemic, and anti-hyperglycemic effects (Getahun, 1976). Various parts of the plant—particularly berries, branches, and leaves—have long been used in traditional medicine to treat a range of ailments (Alipour et al., 2014). Extracts and essential oils from MC leaves and berries have demonstrated inhibitory effects against several pathogenic bacteria (Aleksic and Knezevic, 2014). Moreover, MC leaves and fruits are widely processed for essential oil production and are known to possess strong antimicrobial activity (Usai et al., 2015). Several studies have confirmed the antibacterial potential of MC berries (Cottiglia et al., 2012; Messaoud et al., 2012; Besufekad et al., 2017), and MC-derived compounds have shown significant anti-biofilm properties (Khaleghi and Khorrami, 2021; Khadraoui et al., 2024). Notably, the antimicrobial effects of MC essential oils -including activity against both Gram-positive and Gram-negative bacteria, as well as yeasts and filamentous fungi -are among the most extensively investigated (Mansouri et al., 2001; Kordali et al., 2016; Sharifazizi et al., 2017; Yangui et al., 2017).

Despite the well-documented bioactivity of MC leaf extracts (MCLE), their potential effectiveness against *X. fastidiosa* has not yet been evaluated. This study addresses this knowledge gap by presenting, for the first time, a detailed investigation of the antimicrobial effects of MC and its lyophilized leaf extract (MCLE) against Xfp, Xfm, and Xff, using both *in vitro* and *in planta* approaches.

## 2 Materials and methods

### 2.1 Xylella fastidiosa subspecies

Three *X. fastidiosa* (Xf) subspecies were used in both *in vitro* and *in planta* assays: Xfp sequence type (ST)53, originally isolated from olive (*Olea europaea*); Xfm ST26, first isolated from almond (*Prunus dulcis*) (Montilon et al., 2024); and Xff ST1, recently recovered from almond in Apulia. All subspecies were stored at –80°C in Pierce’s Disease 3 (PD3) broth supplemented with 50% glycerol. When required, cryopreserved cultures of Xfp were plated on buffered charcoal yeast extract (BCYE) medium (Wells et al., 1981), while Xfm and Xff were cultured on PD3 medium (Davis, 1980).

### 2.2 Growth conditions of *Nicotiana benthamiana* plants

*Nicotiana benthamiana* was selected as the model plant for the experiment. Plants were grown under controlled greenhouse conditions in 0.8 L pots, maintained at 25 ± 2°C during the day and 18 ± 2°C at night, with a relative humidity of 60% (Baró et al., 2022). Irrigation was carried out every three days, and 1 g of NPK fertilizer (15-9-15) was applied monthly. To prevent insect infestations, appropriate pest management treatments were applied as needed. Plants were used for inoculation assays at four weeks of age.

### 2.3 Production and metabolite characterization of MCLE

Leaves of *M. communis* (MC) biotype “Tarantino” were selected due to their well-documented antibacterial properties (Hennia et al., 2018). Specifically, freshly collected leaves from a mature shrub located in the municipality of Laterza (TA, Apulia, Italy; 40.631303° N, 16.798915° E) were washed with cold running water and air-dried on absorbent paper. The dried leaves were then ground using a laboratory mixer, and the extraction was performed by adding Milli-Q water at a 1:20 (w/v) ratio. The mixture was subjected to sonication for 5 minutes, followed by centrifugation at 10,000 rpm for 5 minutes. The resulting supernatant was filtered through Whatman filter paper to obtain the crude aqueous extract, then freeze-dried obtaining 5.45 g of dried extract from 100 g of leaves. The metabolite profiling of the extract was carried out using an Ultra-High-Performance Liquid Chromatography (UHPLC) system coupled with a Quadrupole-Orbitrap hybrid mass spectrometer (Q Exactive™ Orbitrap MS/MS; Thermo Fisher Scientific, Bremen, Germany) equipped with an electrospray ionization (ESI) source. The lyophilized MCLE extract was dissolved in a water/methanol solution (9:1, v/v) to a final concentration of 0.5 mg/mL. Chromatographic separation was performed on a Kinetex EVO C18 column (5 μm, 150 mm × 2.1 mm; Phenomenex, Aschaffenburg, Germany). The mobile phases consisted of (A) 0.1% formic acid in water and (B) 0.1% formic acid in acetonitrile. The gradient elution was applied at a flow rate of 0.2 mL/min as follows: 0–11 min: 10–43% B; 11–25 min: 43–61% B; 25–38 min: 61–100% B; 38–41 min: held at 100% B; 41–42 min: 100–10% B; 42–47 min: re-equilibration at 10% B.

The mass spectrometer operated in negative ion mode, with 10 μL of sample injected per run. ESI parameters were set as follows: ion source temperature 300 °C, sheath gas flow (N_2_) 50 units, auxiliary gas flow 10 units, and sweep gas flow 0 units. The MS scan range was set to m/z 150–1400. For MS/MS analyses, a data-dependent acquisition method was employed to fragment precursor ions corresponding to the most intense peaks, using a normalized collision energy of 30%.

Data acquisition, instrument control, and data processing were performed using Xcalibur software (version 2.2, Thermo Fisher Scientific). Metabolites were tentatively identified based on accurate m/z values, predicted molecular formulas, and MS/MS fragmentation patterns, and were compared against published literature and online databases such as MassBank (https://massbank.eu/MassBank/, accessed on 24 April 2025) and FoodB (https://foodb.ca/, accessed on 24 April 2025).

### 2.4 Antimicrobial activity of MCLE against Xfp, Xfm and Xff *in vitro*

#### 2.4.1 Well diffusion test

For the agar well diffusion assay, plates containing 20 mL of BCYE agar medium were uniformly seeded with a Xf suspension arranged in three rows. A single well (8 mm in diameter) was created at the top of the central row and filled with 150 μL of MCLE at four different dilutions: 1:5 (1 × 10^5^ ppm), 1:10 (5 × 10^4^ ppm), 1:25 (2 × 10^4^ ppm), and 1:100 (5 × 10³ ppm) (Zicca et al., 2020). Ampicillin (50 ng/mL) was used as the positive control. All treatments were performed in triplicate. Plates were incubated at 28°C for at least 6 days, after which antimicrobial activity was assessed by measuring the inhibition zone, defined as the distance between the edge of the well and the nearest visible Xf growth.

#### 2.4.2 Broth dilution test

For the broth dilution assay, 20 mL of PD3 broth were dispensed into sterile 50 mL tubes. Each tube was inoculated with 200 μL of an Xf suspension (10^8^ CFU/mL) and supplemented with 150 μL of (MCLE) at different dilution ratios (1:5, 1:10, 1:25, 1:100), corresponding to final concentrations of 740, 370, 140, and 37 ppm, respectively. Control treatments were included as follows: tubes containing only MCLE at the respective dilutions (without bacterial inoculum) served as blanks, while tubes inoculated with Xf in the absence of MCLE served as positive controls. Following inoculation, all tubes were incubated at 28°C for 14 days. Bacterial growth was monitored spectrophotometrically every two days by measuring optical density at 600 nm (BECKMAN DU-640, Brea, California, USA).

### 2.5 Toxicity evaluation of MCLE

The potential phytotoxicity of MCLE was evaluated on *N. benthamiana*. Using a 0.5 mL insulin syringe, 50 μL of MCLE at different concentrations (1 × 10^5^, 5 × 10^4^, 2 × 10³, and 1.5 × 10² ppm) were infiltrated into fully expanded leaves. Three infiltrations were performed per plant, with a total of six replicates. Sterile distilled water was infiltrated in place of MCLE and served as the negative control. Plants were maintained in a greenhouse at 25 ± 3°C for at least 48 h. Phytotoxicity was evaluated through visual observation of symptoms and direct measurement of lesion diameter at the infiltration sites. Additionally, a scoring system was employed to assess lesion severity on a scale from 0 to 3 (0 = absent, 1 = mild, 2 = moderate, 3 = severe).

### 2.6 *In planta* antagonistic activity of MCLE against Xf

**Figure 1:**
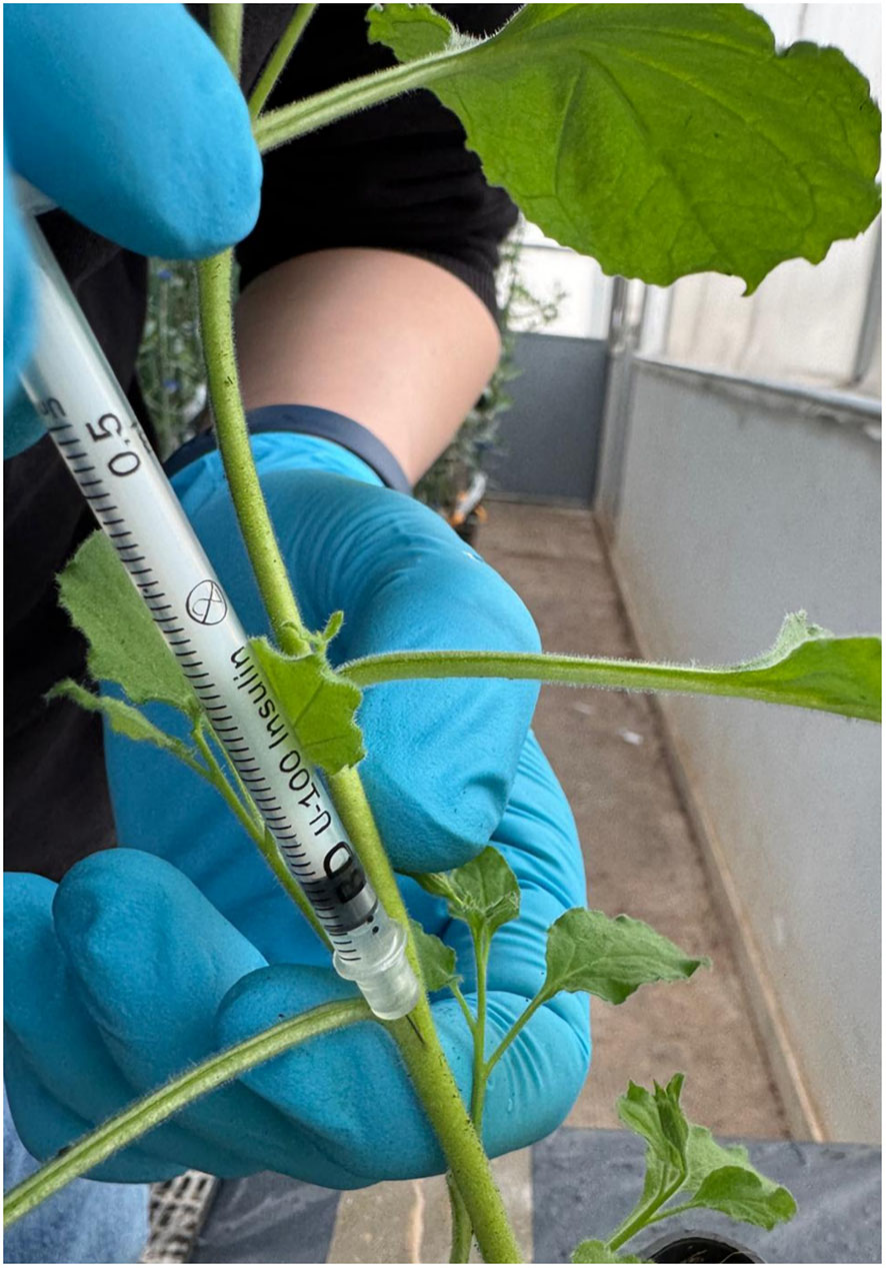
Needle inoculation of *Xylella fastidiosa* subspecies in *Nicotiana benthamiana* plants.

An *in planta* assay was conducted to evaluate the antagonistic activity of MCLE against Xf subspecies within plant tissues. The experiment involved Xf inoculation and subsequent application of MCLE to the stems of *N. benthamiana*, a validated model host for Xf (Baró et al., 2022). One-month-old *N. benthamiana* plants were inoculated using a 0.5 mL hypodermic syringe, with the needle inserted approximately halfway into the stem diameter to reach the vascular system (**Figure 1**). Bacterial suspensions were prepared at 10^8^ CFU/mL, corresponding to an OD_600_ of 0.5 for Xfp and Xfm, and an OD_600_ of 0.3 for Xff. Two inoculations (15 μL each) of Xf suspension in PBS were administered at two distinct stem positions, 8 and 12 cm above the soil surface, for a total inoculum of 3 × 10^6^ CFU/mL per plant. MCLE applications were performed on the same side of the stem, with two treatments of 15 μL each (1,500 ppm) at 6 and 14 cm above the soil surface. Each treatment group consisted of seven plants, including both positive and negative controls for all subspecies tested. All inoculated plants were maintained under controlled quarantine greenhouse conditions at 25 ± 3°C.

### 2.7 DNA extraction and qPCR assay for *in planta* detection and quantification of Xf subspecies

Genomic DNA was extracted from 500 mg of *N. benthamiana* leaf petioles after homogenization in extraction bags (Bioreba AG, Reinach, Switzerland) using a Homex apparatus (Bioreba AG), following a CTAB-based protocol (Doyle and Doyle, 1987). DNA quality and concentration were assessed with a NanoDrop 2000 spectrophotometer (Thermo Fisher Scientific Inc., Wilmington, DE, USA). Quantitative PCR (qPCR) assays were performed to confirm infection by Xf subspecies in inoculated plants, using primers and conditions described by Harper et al. (2010). Assays were conducted at 20 and 40 days post-inoculation (dpi) to monitor infection progression. All reactions were run in triplicate and included a negative control (NC), positive control (PC), and no-template control (NTC), along with subspecies-specific standard curves. Standard curves were generated from cell suspensions of Xfp, Xfm, and Xff at concentrations ranging from 10^7^ to 10¹ CFU/mL. For Xfp and Xfm, the starting suspension corresponded to an OD_600_ of 0.5 (~10^8^ CFU/mL), whereas for Xff an OD_600_ of 0.3 was used. These curves were employed to quantify bacterial loads in the infected plant samples.

### 2.8 Statistical analysis

To evaluate the significance of Xfp, Xfm, and Xff growth inhibition, a one-way ANOVA was performed using CoStat software, version 6.451 (Cohort, Monterey, CA, USA). For the broth dilution assay, data were analyzed by two-way ANOVA followed by Tukey’s *post hoc* test to identify statistically significant differences (p < 0.05). In addition, to assess the *in planta* effect of the extract on Xf subspecies, quantification cycle (Cq) values were calculated, and a one-way ANOVA was applied using the same software.

## 3 Results

### 3.1 Metabolite profile of MCLE

MCLEs were analyzed by LC-ESI-Q Exactive MS in both negative and positive ion modes to maximize metabolite coverage and obtain high-resolution MS and MS/MS spectra. The negative ion LC-MS profile revealed the predominance of phenolic acids, flavonol derivatives, and ellagitannins. A total of 35 major metabolites were detected, of which 25 were putatively identified based on accurate m/z values, predicted molecular formulas, and MS/MS fragmentation patterns (**Table 1**). Most of these compounds have previously been reported in MC leaves (Taamalli et al., 2014; D’Urso et al., 2019). The main groups detected in negative ion mode included ellagitannins (e.g., galloyl hexose derivatives), flavonols (mainly myricetin, quercetin, and kaempferol derivatives), and phenolic acids.

**Table 1:** *Myrtus communis* biotype “Tarantino” leaf extract: UHPLC-ESI-Q-Exactive-MS and UHPLC-ESI-Q-Exactive-MS/MS analysis in negative ion mode.

N°	Rt	[M-H]^-^	Molecular formula	Δppm	MS/MS	Identity	Reference
1	1.89	383.1198	C_14_H_23_O_12_	3.8	191	unknown	
2	1.89	643.1735	C_24_H_35_O_20_	3	83.0/175.0/217.0/113.0	unknown	
3	2.19	643.1740	C_24_H_35_O_20_	3.8	83.0/175.0/217.0/113.0	unknown	
4	2.19	741.1514	C_31_H_33_O_21_	0.8	175.0/217.0	unknown	
5	2.19	835.2022	C_48_H_35_O_14_	0.2	191	unknown	
6	3.14	633.0737	C_27_H_21_O_18_	2.5	301.0/275.0/249.0	strictinin isomer 1	D’Urso et al., 2019
7	3.14	483.0786	C_20_H_19_O_14_	3.5	169.0/332.1/313.1	1,6-di-*O*-galloylglucose	Mass Bank
8	3.26	343.0662	C_14_H_15_O_10_	0.8	191	galloylquinic acid	D’Urso et al., 2019
9	4.07	633.0741	C_27_H_21_O_18_	2.9	301.0/275.0/249.0	strictinin isomer 2	D’Urso et al., 2019
10	4.07	401.1092	C_17_H_21_O_11_	3.5	265.1/295.1/277.1	4-Hydroxy-5-(3',5'-dihydroxyphenyl)-valeric acid-O-glucuronide	FoodB
11	5.19	807.0615	C_25_H_27_O_30_	3.3	301.0/261.0/169.0	unknown	
12	6.53	782.0593	C_34_H_22_O_22_	-0.5	301.0/275.0/451.0	punicalin	D’Urso et al., 2019
13	6.53	361.0777	C_14_H_17_O_11_	3.3	249.1/111.0	unknown	
14	7.28	495.0783	C_21_H_19_O_14_	2.8	169.0/191.1/343.1	digalloylquinic acid	D’Urso et al., 2019
15	7.45	783.0679	C_34_H_24_O_22_	0.5	301.0/275.0/765.1	cornusin	D’Urso et al., 2019
16	8.23	859.0776	C_25_H_31_O_33_	4	301.0/765.0	unknown	
17	8.7	431.1923	C_20_H_31_O_10_	2.6	101.0/59.0/161.0/269.1	neorehmannioside	Taamalli et al., 2014
18	8.7	451.1250	C_21_H_23_O_11_	3.4	169.0/313.1	aspalathin	MassBank
19	9.03	431.1925	C_20_H_31_O_10_	3.1	285	vitexin	D’Urso et al., 2019
20	9.06	647.0903	C_28_H_23_O_18_	3.7	169.0//343.1/495.1	trigalloylquinic acid	D’Urso et al., 2019
21	9.56	631.0948	C_28_H_23_O_17_	2.9	316.0/479.1	myricetin galloyl hexoside	D’Urso et al., 2019
22	10.05	479.0836	C_21_H_19_O_13_	3.3	316	myricetin galactoside	D’Urso et al., 2019
23	10.19	371.0986	C_16_H_19_O_10_	3.7	121.0/.249.1	deacetylasperuloside	Crescenzi et al., 2021
24	10.35	305.0705	C_15_H_13_O_7_	3.5	225.1	epigallocathechin	Taamalli et al., 2014
25	10.35	447.0576	C_20_H_19_O_12_	3.9	300	quercetin rhamnoside	D’Urso et al., 2019
26	10.6	615.1008	C_28_H_23_O_16_	4.4	300.0/463.1	myricetin galloylrhamnopyranoside	D’Urso et al., 2019
27	10.85	463.0883	C_21_H_19_O_12_	2.6	316	ellagic acid hexoside	D’Urso et al., 2019
28	11.57	533.1312	C_25_H_25_O_13_	4.2	251.1/281.1/413.1/443.1	6,8-di-C-β-D-arabinopyranosylapigenin	FoodB
29	11.84	447.0939	C_21_H_19_O_11_	3.8	300	quercetin rhamnoside	D’Urso et al., 2019
30	12.63	493.2299	C_22_H_37_O_12_	3.2	315.2/161.0/131.0	zingiberoside	Negm et al., 2022
31	12.99	571.24.04	C_27_H_39_O_13_	3.7	165.0/195.1/345.1	unknown	
32	13.39	567.2086	C_27_H_35_O_13_	2.5	271.0/169.0/313.1	gallomyrtucommulone C	D’Urso et al., 2019
33	14.01	567.2081	C_27_H_35_O_13_	1.6	271.0/169.0/313.1	gallomyrtucommulone C	D’Urso et al., 2019
34	14.23	569.2242	C_27_H_37_O_13_	2.3	271.0/169.0/313.1	gallomyrtucommulone A	D’Urso et al., 2019
35	15.64	327.2188	C_18_H_31_O_9_	4.8	171.1/211.1/229.1	trihydroxy-octadecaedienoic acid	Crescenzi et al., 2021

Metabolites annotated at level 2 based on accurate m/z values, predicted molecular formulas, and MS/MS fragmentation patterns (Sumner et al., 2007).

The positive ion LC-MS profile likewise confirmed the presence of phenolic acids and flavonol derivatives, particularly caffeoylquinic acids and myricetin derivatives. In this mode, 19 major metabolites were detected, of which 12 were putatively identified using accurate m/z values, molecular formulas, and fragmentation patterns (**Table 2**). Notably, compound 13′, secoisolariciresinol-9-glucoside, was identified for the first time in MC leaves.

**Table 2:** *Myrtus communis* biotype “Tarantino” leaf extract: HPLC-ESI-Q-Exactive-MS and UHPLC-ESI-Q-Exactive-MS/MS analyses in positive ion mode.

N°	Rt	[M+H]^+^	Molecular formula	Δppm	MS/MS	Identity	Reference
1’	2.45	332.1342	C_14_H_22_O_8_N	0.6	108.1/152.1/314.1	unknown	
2’	7.27	479.0816	C_21_H_19_O_13_	-0.7	153	quercetin-3-*O*-glucuronide	Mass Bank
3’	7.71	355.1022	C_16_H_19_O_9_	-0.4	193	caffeoyl quinic acid	Mass Bank
4’	8.18	355.1022	C_16_H_19_O_9_	-0.4	193	caffeoyl quinic acid	Mass Bank
5’	9.05	425.1575	C_24_H_25_O_7_	-4.6	263.1	unknown	
6’	9.54	633.1088	C_28_H_25_O_17_	0.3	319.0/153.0	myricetin galloyl hexoside	D’Urso et al., 2019
7’	10.01	319.0446	C_15_H_11_O_8_	-0.8	273.0/245.0/153.0	myricetin	MassBank
8’	10.01	481.0976	C_21_H_21_O_13_	-0.2	319	myricetin-3-*O*-galactoside	MassBank
9’	10.26	383.1334	C_18_H_23_O_9_	-0.5	263.1//347.1/233.0/317.0	unknown	
10’	10.26	593.2204	C_29_H_37_O_13_	-4.2	203.0/234.1/383.1/413.1	unknown	
11’	10.86	319.0442	C_15_H_11_O_8_	-4.2	273.0/245.0/153.0	myricetin	MassBank
12’	10.86	465.1026	C_21_H_21_O_12_	-0.4	303.0/319.0	myricetin-3-*O*-rhamnoside	MassBank
13’	11.33	525.2311	C_26_H_37_O_11_	-3.7	137.1/163.1/393.2	secoisolariciresinol 9-glucoside	FoodB
14’	11.58	383.1340	C_18_H_23_O_9_	0.9		unknown	
15’	11.86	303.0499	C_15_H_11_O_7_	0.1	257.0/285.0/229.0	quercetin	Taamalli et al., 2014
16’	11.86	449.1079	C_21_H_21_O_11_	0.3	287.1	luteolin glucoside	Taamalli et al., 2014
17’	13.43	551.2124	C_27_H_35_O_9_	0.2	193.2/221.1	unknown	
18’	13.43	439.1939	C_22_H_31_O_9_	-5.3	203.0/209.1/277.1	unknown	
19’	13.77	545.1629	C_27_H_29_O_12_	-4.5	317.1/365.3/383.3	unknown	

Metabolites annotated at level 2 based on accurate m/z values, predicted molecular formulas, and MS/MS fragmentation patterns (Sumner et al., 2007).

### 3.2 *In vitro* antimicrobial activity of MCLE against Xfp, Xfm *and* Xff

#### 3.2.1 Well diffusion test

Four MCLE dilutions (1:5, 1:10, 1:25, 1:100) were tested for their ability to inhibit Xfp, Xfm, and Xff using the *in vitro* well diffusion assay. The results demonstrated that MCLE exhibited antimicrobial activity against all three *Xylella* subspecies (**Table 3**).

**Table 3:** Antimicrobial activity of *Myrtus communis* leaf extract against *Xylella fastidiosa* subspecies (*pauca*, *multiplex*, and *fastidiosa*).

Subspecies	Host plant	Plant extract tested	Dilution	Growth inhibition (mm)	Std deviation
*Xylella fastidiosa* subsp. *pauca*	*Olea europaea*	*Myrtus communis*	1/5	22.6 ± 0.2	± 2.5^b^
			1/10	9.3 ± 0.3	± 1.5^c^
			1/25	6.6 ± 0.2	± 0.5^c^
			1/100	–	–
		Ampicillin	50 ng/ml	33.0 ± 0.4	± 2^a^
*Xylella fastidiosa* subsp. *multiplex*	*Prunus dulcis*	*Myrtus communis*	1/5	4.8 ± 0.3	± 1.1^b^
			1/10	–	–
			1/25	–	–
			1/100	–	–
		Ampicillin	50 ng/ml	28.0 ± 0.2	± 2^a^
*Xylella fastidiosa* subsp. *fastidiosa*	*Prunus dulcis*	*Myrtus communis*	1/5	7.3 ± 0.4	± 1.0^b^
			1/10	–	–
			1/25	–	–
			1/100	–	–
		Ampicillin	50 ng/ml	29.3 ± 0.2	± 1.2^a^

(-) No inhibition halo, Different letters were used to indicate statistically different inhibitory activity of the extracts with p < 0.05 as determined by one-way analysis of variance (ANOVA) followed by Tukey’s test.

**Figure 2:**
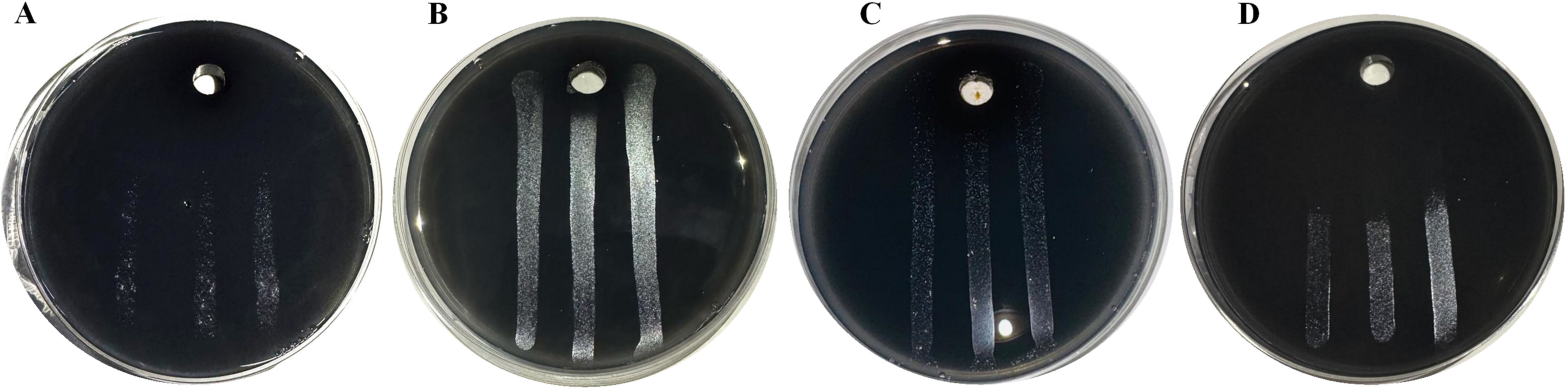
Antimicrobial activity of *Myrtus communis* biotype “Tarantino” leaf extract at 1:5 dilution against *Xylella fastidiosa* subspecies: *pauca***(A)**, *multiplex***(B)**, and *fastidiosa***(C)**. Panel **(D)** shows positive control with ampicillin (50 ng/mL).

The highest concentration (1:5; 1 × 10^5^ ppm) strongly suppressed Xfp growth, producing large inhibition halos with a mean diameter of 22.6 mm (**Figure 2**). In contrast, the 1:10 and 1:25 dilutions produced significantly (p < 0.05) smaller zones of inhibition, measuring 9.3 mm and 6.6 mm, respectively.

By comparison, the same 1:5 dilution generated much smaller inhibition zones for Xfm (4.8 mm) and Xff (7.3 mm). None of the lower concentrations (1:10, 1:25, 1:100) displayed measurable antimicrobial activity against either Xfm or Xff.

#### 3.2.2 Broth dilution test

**Figure 3:**
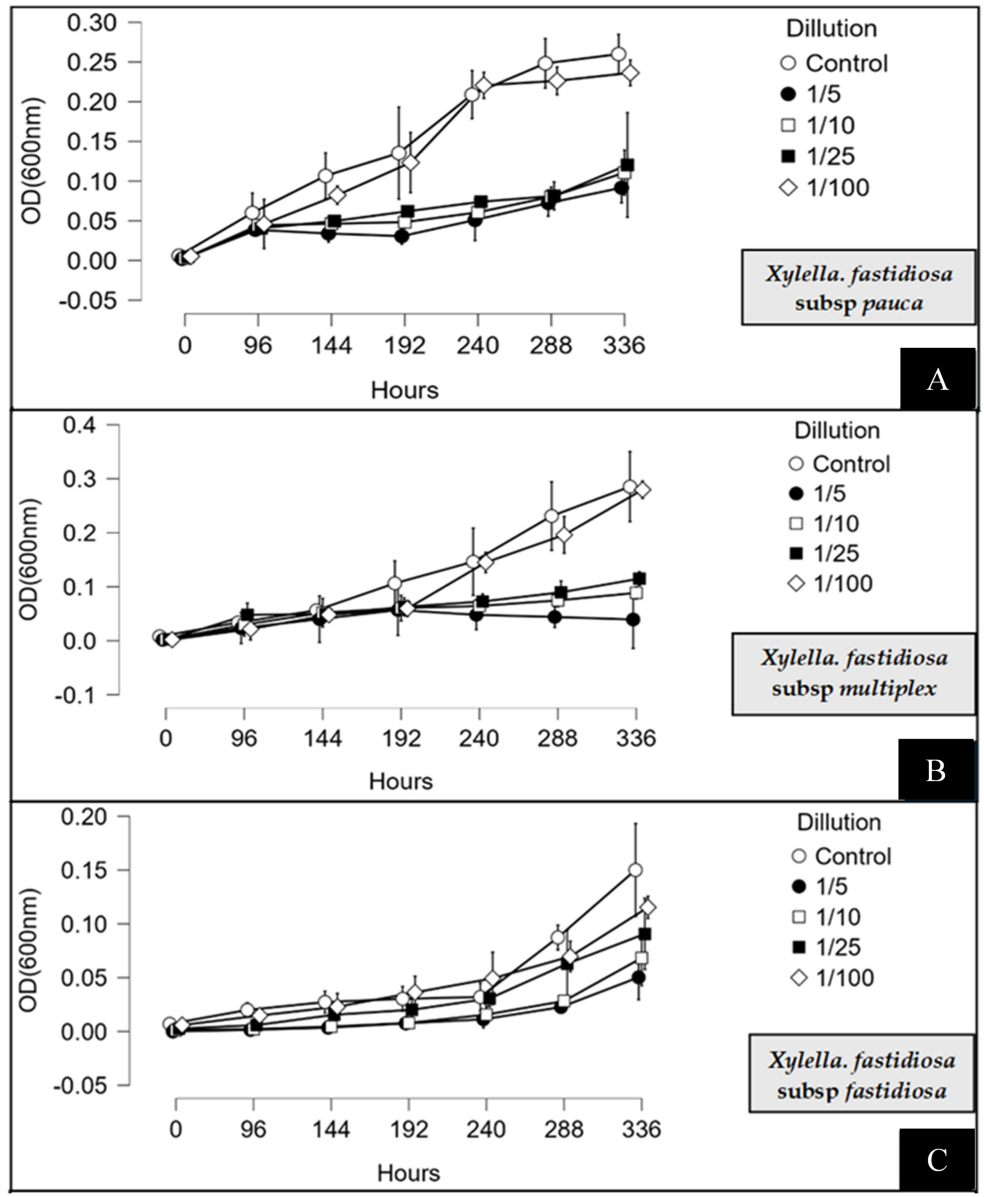
Antimicrobial activity of *Myrtus communis* biotype “Tarantino” leaf extract at different dilutions (1:5, 1:10, 1:25, and 1:100) against *Xylella fastidiosa* subsp. *pauca***(A)**, *fastidiosa***(B)**, and *multiplex***(C)**. The extract dilutions were added individually to PD3 broth, inoculated with the respective subspecies, and bacterial growth was monitored spectrophotometrically at A600 over a 14-day incubation period. The bars indicate the error means for three replicates.

The inhibitory effect of MCLE on Xf growth in PD3 liquid medium was monitored over a 14-day incubation period. A marked reduction in OD_600_ was observed for all three subspecies compared with the untreated controls. Specifically, at the 1:5 dilution, Xfp growth after 336 h was reduced to OD_600_ = 0.091 ± 0.001 compared with OD_600_ = 0.260 ± 0.003 in the control, corresponding to 65% growth inhibition (**Figure 3**).

A similar inhibitory effect was observed for Xfm and Xff. After 336 h, Xfm growth was reduced to OD_600_ = 0.039 ± 0.002 versus 0.282 ± 0.001 in the control, corresponding to 86% inhibition. For Xff, growth was reduced to OD_600_ = 0.046 ± 0.001 compared with 0.154 ± 0.004 in the control, equivalent to 66% inhibition. Notably, growth reduction was detectable as early as 96 h post-inoculation, with inhibition levels generally increasing over time. At lower concentrations, inhibition was less pronounced. For example, the 1:100 dilution produced only slight reductions, with OD_600_ values of 0.23 ± 0.001, 0.11 ± 0.002, and 0.27 ± 0.002 for Xfp, Xff, and Xfm, respectively, compared with their corresponding controls after 336 h. Overall, Xf growth was strongly inhibited at 1:5, 1:10, and 1:25 dilutions, with inhibition levels directly proportional to extract concentration across all three subspecies. These findings demonstrate that MCLE effectively suppresses Xf growth in liquid culture, supporting its potential application in Xf control (**Figure 3**).

### 3.3 Toxicity evaluation of MCLE

**Figure 4:**
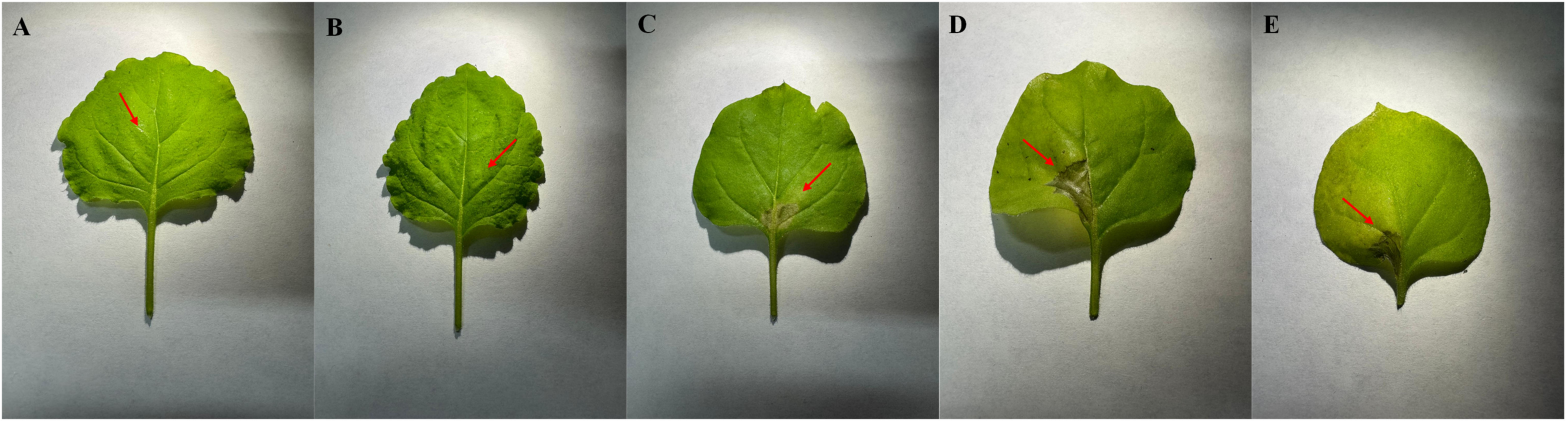
Toxicity assay of *Myrtus communis* biotype “Tarantino” leaf extract on *Nicotiana benthamiana* leaves. Treatments: **(A)** water control, **(B)** 1,500 ppm, **(C)** 20,000 ppm, **(D)** 50,000 ppm, and **(E)** 100,000 ppm.

The potential phytotoxicity of MCLE was evaluated on *N. benthamiana* leaves to assess its suitability as an eco-friendly candidate for Xf control. At 1,500 ppm, no hypersensitive reactions were observed (0 score). At 20,000 ppm, however, a mild hypersensitive response developed, characterized by halo-shaped tissue necrosis approximately 8 mm in diameter (data not shown). Higher concentrations (50,000 ppm and 100,000 ppm) induced a strong hypersensitive reaction, producing dark halo-shaped necrotic lesions (3 score, **Figure 4**). These results provide preliminary evidence supporting the possible safe use of MCLE at lower concentrations. Nonetheless, further evaluations are required to confirm its safety and effectiveness across different host plants and environmental conditions.

### 3.4 Assessment of MCLE on symptom development of Xf in *N. benthamiana*

**Figure 5:**
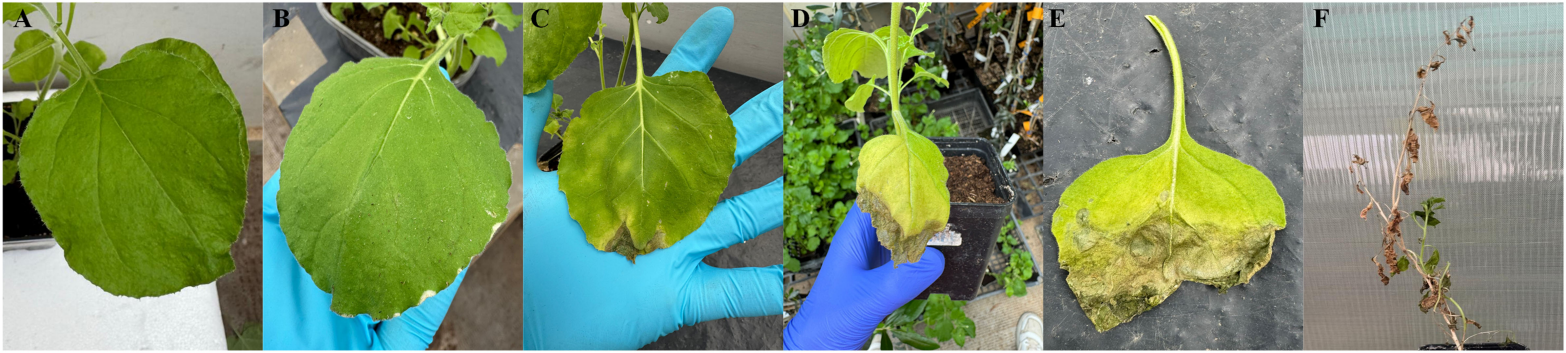
Progression of symptom severity on *Nicotiana benthamiana* leaves, illustrated according to a visual scale from 0 (no visible symptoms) to 5 (severe symptoms), panels **(A–F)**, respectively.

The effect of MCLE on the development of Xf symptoms in *N. benthamiana* plants was assessed under controlled conditions. In the positive control group, the first symptoms appeared at 30 days post-inoculation (dpi) (**Figure 5**). These early manifestations differed from the typical leaf scorch associated with Xf infection; instead, a characteristic rounding of apical leaves was observed across all subspecies-inoculated controls at 30 dpi. Classical leaf scorch symptoms became evident only after 60 dpi. For all subspecies tested, symptom development followed a similar pattern, characterized by progressive leaf scorching followed by plant wilting. Importantly, symptoms were initially observed only in plants inoculated with Xf.

To standardize symptom assessment, a visual scale ranging from 0 to 5 was adapted from that described for grapevines (Clifford et al., 2013): 0 = asymptomatic leaves; 1 = mild symptoms; 2 = clear symptoms; 3 = moderate leaf scorch; 4 = severe, widespread leaf scorch; 5 = dead plant (**Figure 5**). Symptom progression was consistent across all Xf subspecies.

### 3.5 qPCR assay for *in planta* detection of Xf

Quantitative PCR (qPCR) assays were performed to assess the bacterial load of Xf in *N. benthamiana* plants. An initial assay was carried out 18 days post-inoculation (dpi) to verify successful infection. For this purpose, a pool of four plants was tested to confirm the presence of Xf. Subsequently, two additional time points were selected to monitor bacterial colonization: 20 dpi and 40 dpi.

**Figure 6:**
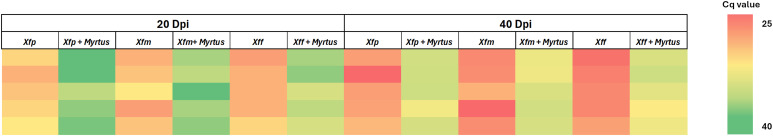
Heat map showing real-time PCR quantification of treated and untreated *Nicotiana benthamiana* plants. Data are presented as mean Cq values from non-inoculated controls and plants inoculated with *Xylella fastidiosa* subsp. *pauca*, *multiplex*, and *fastidiosa*.

For each assay, petioles were collected from leaves at different heights above the inoculation site. Standard curves for all three subspecies were included in each run. Quantification cycle (Cq) values were recorded for all samples. At both time points, plants inoculated with Xf but not treated with MCLE exhibited significantly lower Cq values (F = 120; p < 0.001), reflecting higher bacterial loads compared with MCLE-treated plants. These differences are clearly illustrated in the heat map (**Figure 6**).

**Figure 7:**
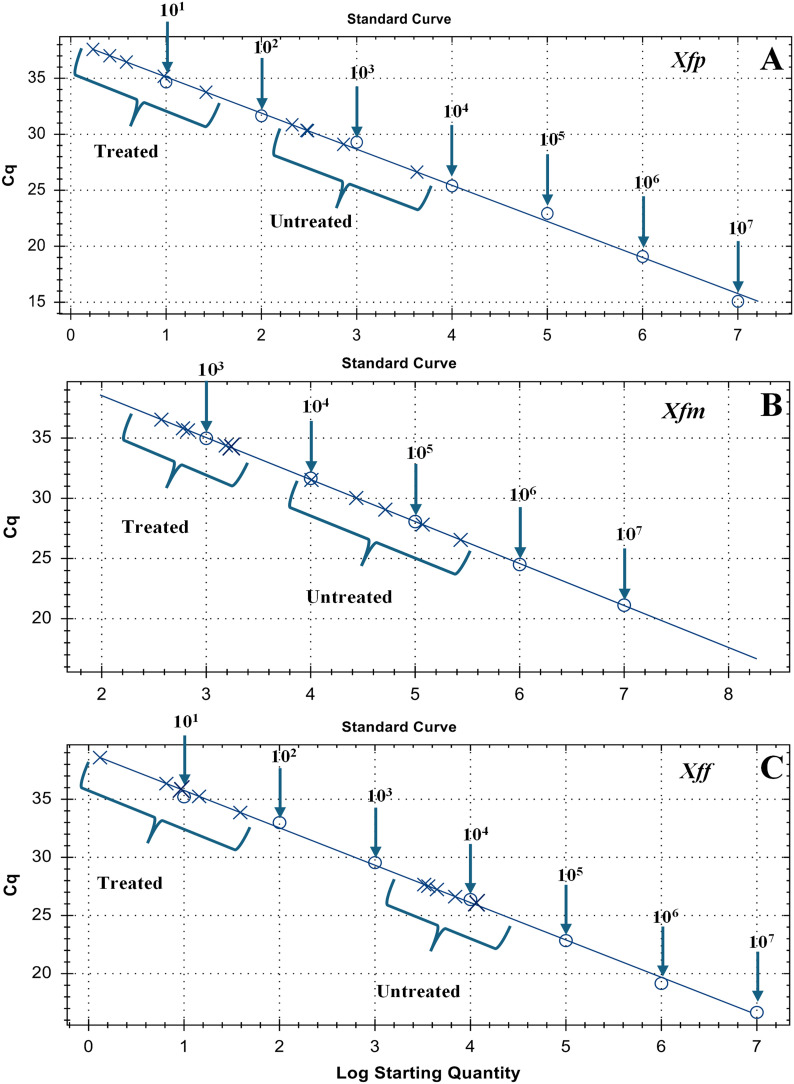
qPCR standard curves showing differences between untreated and treated plants inoculated with *Xylella fastidiosa* subsp. *pauca*, *multiplex*, and *fastidiosa*. Subsp. pauca (A), multiplex (B) and fastidiosa (C).

Moreover, qPCR results showed that treated samples consistently clustered in regions of the standard curve corresponding to low bacterial concentrations, a trend observed across all three subspecies (**Figure 7**). Specifically, for Xfp, treated samples clustered around 10¹ CFU, whereas untreated plants ranged between 10³ and 10^4^ CFU. For Xfm, treated plants reached approximately 10³ CFU compared with 10^5^ CFU in untreated plants. The most pronounced effect was observed for Xff, where treated plants consistently showed ~10¹ CFU, in contrast to 10^5^ CFU in untreated controls.

## 4 Discussion

The emergence of *Xylella fastidiosa* (Xf) poses major challenges to global agriculture, threatening economically important crops and leading to high yield losses, shifts in cultivation practices, and potential long-term damage to production systems. This pathogen has an exceptionally broad host range, colonizing over 500 plant species across 85 families (Baró et al., 2022). Notably, in Europe it has devastated olive orchards in the Apulia region of Italy (the olive quick decline syndrome), prompting the planting of resistant varieties and other drastic measures. In this context, there is an urgent need for sustainable, effective control strategies that comply with regulatory constraints (e.g. the prohibition of antibiotic use in agriculture in the EU). Plant extracts and plant-derived compounds are increasingly recognized as valuable resources in managing diseases caused by bacterial and fungal pathogens (Portaccio et al., 2025). Such natural products align with the principles of sustainable agriculture and integrated pest management, offering broad-spectrum activity and a lower likelihood of inducing resistance compared to conventional bactericides.

Among these, extensive studies have documented the antimicrobial activity of *Myrtus communis* (myrtle) leaf extracts against a wide range of plant and human pathogens. Building on this evidence, the present study investigated the antimicrobial activity of *M. communis* biotype “Tarantino” leaf extract (MCLE) against three Xf subspecies (Xfp, Xfm, and Xff) through both *in vitro* and *in planta* assays. Our results clearly demonstrated that MCLE possesses antibacterial properties against all the three subspecies of Xf. This is consistent with prior reports of myrtle’s broad antimicrobial effects (Aleksic and Knezevic, 2014; Mahmoudvand et al., 2015; Besufekad et al., 2017; Cordeiro et al., 2020). Such broad-spectrum efficacy highlights the potential of MCLE as a natural antibacterial compound.

Different parts of the myrtle plant are rich in diverse bioactive compounds, including phenolics, terpenoids, and flavonoids (Appendino et al., 2002; Tuberoso et al., 2006; Yoshimura et al., 2008). Chemical profiling by LC-MS in our study highlighted the predominance of phenolic acids, flavonol derivatives (mainly myricetin, quercetin, and kaempferol conjugates), and ellagitannins (e.g. galloyl-hexose derivatives) in the MCLE biotype Tarantino, confirming for most of them previous findings (Taamalli et al., 2014; D’Urso et al., 2019). Hydrolysable tannins (ellagitannins) and flavonoids, such as myricetin and quercetin glycosides, as well as other metabolites are known for limiting *X. fastidiosa* growth (Maddox et al., 2010; Bleve et al., 2018; Rongai et al., 2024). These phenolic compounds are known to exert antimicrobial effects through multiple mechanisms. For instance, flavonoids, like catechin, stilbenes, and resveratrol, can disrupt bacterial cell membranes, and phenolic acids, such as gallic acid and epicatechin, interfere with bacterial adhesion by downregulating genes for adhesive structures (e.g. *fimA*, *xadA*) (Lee et al., 2020). This inhibition of adhesion and biofilm formation is crucial, as biofilms significantly contribute to bacterial tolerance against antimicrobial treatments. Remarkably, we also identified secoisolariciresinol-9-glucoside—a glycosylated lignan—in MCLE, marking the first report of this compound in myrtle. This lignan is noteworthy due to its reported biological activities, including antioxidant and antimicrobial potential. Its MS/MS fragmentation pattern it has been described in several plant species, such as *Moringa oleifera* and various *Ribes* spp (Razgonova et al., 2024; Gao et al., 2025). Lignans such as secoisolariciresinol have also been reported for their antimicrobial activity against human-associated pathogens, including *Staphylococcus aureus*, *Pseudomonas aeruginosa*, *Escherichia coli*, *Candida albicans*, and *Aspergillus brasiliensis* (Kyselka et al., 2017). The identification of this compound in myrtle paves the way for further research into its activity against emerging plant pathogens such as Xf.

Consistent with previous reports of myrtle’s antimicrobial activity (Aleksic and Knezevic, 2014; Besufekad et al., 2017; Cordeiro et al., 2020), our results confirmed strong inhibitory effects of MCLE *in vitro*. In agar well-diffusion assays, the highest concentration tested (1:5 dilution, ~1×10^5^ ppm) produced the greatest inhibition against Xfp, with a mean inhibition zone of 22.6 mm. At the same concentration, clear inhibition halos of 4.8 and 7.3 mm were observed against Xfm and Xff, respectively. Notably, none of the lower concentrations (1:10, 1:25, 1:100) produced measurable inhibition zones against Xfm or Xff in the well-diffusion assays. However, bacterial growth suppression was evident in broth dilution assays, particularly at the higher extract concentrations. These subspecies-specific differences highlight a broader challenge in antimicrobial research: results are often reported using different metrics (minimum inhibitory concentration [MIC], colony-forming units [CFU]/mL reduction, or inhibition zone diameter), which can complicate direct comparisons across studies. Moreover, outcomes vary depending on the experimental method employed—disk diffusion, agar well diffusion, broth microdilution, etc.—as well as the intrinsic properties of the tested compound and the target microorganism (Hennia et al., 2019). For example, Salvagnini et al. (2008) demonstrated that essential oils from Brazilian myrtle exhibited antimicrobial activity against *S. aureus*, *S. epidermidis*, *E. coli*, *Bacillus subtilis*, and *Serratia marcescens*. In their study, agar well-diffusion assays showed greater efficacy than disk diffusion for certain strains (e.g. *S. aureus*, *S. epidermidis*, *B. subtilis*), suggesting that the method of delivery (and consequent contact time and diffusion) can influence the observed activity. This aligns with our observations for Xf and suggests that antimicrobial efficacy is influenced not only by the intrinsic properties of the extract but also by the target organism (even subspecies) and the methodology applied.

Importantly, even the highest dilution of MCLE tested (1:100) produced slight growth inhibition in broth dilution assays for all three Xf subspecies, indicating a dose-dependent effect. This is consistent with previous findings showing concentration-dependent bioactivity of myrtle-derived compounds. For instance, linalool - a key bioactive monoterpene in myrtle leaves - has been shown to inhibit biofilm formation in *P. aeruginosa*, *E. coli*, *Alternaria bambusicola*, and *S. marcescens*. Linalool also suppressed the synthesis of a quorum-sensing-regulated violacein pigment in *Chromobacterium violaceum* by up to 69% at 50 mg/mL, in a dose-dependent manner (Alyousef et al., 2021). The observation that even low concentrations of MCLE can hinder Xf growth to some extent suggests the extract contains potent components active at sub-inhibitory levels. These components may not completely prevent growth at low doses, but they could interfere with critical processes such as cell-cell signaling or adherence, thereby slowing the pathogen’s proliferation.

For Xf, it is crucial to identify plant extracts with antibiofilm activity, given that biofilm formation in xylem vessels is central to this pathogen’s virulence. Xf colonizes host xylem, multiplies, and forms resilient biofilms that occlude water transport, ultimately leading to severe symptoms and host death (Baró et al., 2022; Cardinale et al., 2018). Extracts from various parts of *Myrtus communis* have indeed been investigated as potential sources of antibiofilm compounds (Owlia et al., 2010; Cannas et al., 2014). In the present study, MCLE showed promising antibiofilm effects against Xf *in vitro*, as evidenced by reduced aggregation and sedimentation in liquid culture (data discussed in Results). Our findings are supported by similar studies on myrtle essential oils: for example, Caputo et al. (2022) demonstrated that essential oil from Italian myrtle leaves inhibited biofilm formation and even disrupted mature and ultra-mature biofilms of diverse bacteria (*E. coli* DSM 8579, *P. aeruginosa* ATCC 50071, *Pectobacterium carotovorum* DSM 102074, *S. aureus* DSM 25923, and *Listeria monocytogenes* ATCC 7644). Similarly, myrtenol, a major component of myrtle essential oil, strongly inhibited *S. aureus* biofilm formation at sub-inhibitory concentrations (Cordeiro et al., 2020). These results support MCLE as a promising source of antibiofilm agents against Xf. The proposed mechanisms of action for myrtle-derived compounds include interference with microbial transport proteins and enzymes, disruption of cell wall integrity, inactivation of adhesins needed for surface attachment, and formation of complexes with extracellular polysaccharides that stabilize biofilms (Cai et al., 1989). This agrees with more recent evidence that polyphenols can directly hinder biofilm establishment by Xf. For example, an *in vitro* study showed that a phenolic-rich olive leaf extract (containing oleuropein) significantly inhibited Xf growth and biofilm formation, with oleuropein identified as a particularly potent natural anti-Xf agent (Vizzarri et al., 2023). Such findings illustrate how plant-derived compounds may target the adhesion and matrix components of Xf’s biofilm. However, it should be noted that the composition and architecture of the Xf outer membrane and biofilm matrix remain incompletely understood. Further studies are required to clarify the precise mode of action of MCLE on Xf cells, including which metabolites are responsible for antibiofilm effects and whether they act synergistically. To our knowledge, this is the first report describing the antimicrobial potential of a *Myrtus communis* leaf extract against Xf, highlighting a novel avenue for the control of the pathogen based on natural substances.

To date, the antimicrobial activity of MCLE has been investigated mainly under *in vitro* conditions, while *in planta* studies have been limited. In this study, we performed a series of pathogenicity and treatment assays using *N. benthamiana* as the model host for *Xylella fastidiosa* (Xf). This species is a well-established experimental host, as it develops symptoms relatively rapidly - within a few weeks after inoculation - thereby expediting disease assessment (Francis et al., 2008). Results consistently showed that MCLE treatment reduced symptom severity and delayed disease progression for up to 60 dpi compared with untreated controls, across all three tested Xf subspecies. Importantly, MCLE-treated plants also had lower infection rates (fewer plants became systemically infected) and showed markedly milder symptoms than untreated plants. These protective effects are in line with results reported for other compounds applied against Xf. For instance, N-acetyl cysteine (NAC) – a known disruptor of biofilms – suppressed disease symptoms by 75–80% in Xf-infected sweet orange plants under greenhouse conditions (Muranaka et al., 2013). Similarly, oxytetracycline trunk injection of Xf-infected almond trees reduced disease severity by ~73%, which corresponded to significantly lower bacterial levels as detected by ELISA (Amanifar et al., 2016). Our findings with MCLE are comparable: although symptoms were not eliminated, their onset was delayed and their severity attenuated in treated plants. This further validates *Nicotiana* spp. as practical surrogates to study Xf-host interactions and control measures, complementing more time-consuming tests in natural woody hosts.

In terms of bacterial load reduction, MCLE-treated plants consistently harbored lower Xf populations than untreated controls, corroborating the direct growth-inhibitory effect observed *in vitro*. Quantitative PCR and culturing from petioles revealed that MCLE applications reduced Xf titers by at least 1–3 orders of magnitude (depending on subspecies) relative to controls by 20 dpi. However, a single application of MCLE was insufficient to fully eradicate the infection or to prevent symptom development in the long term. Residual bacterial populations (~10^1–^10^3^ CFU/g of tissue) were still detected in some treated plants after 20 days, and symptoms eventually progressed, although more slowly than in controls. This outcome is like what has been observed in other systems; for example, in Xf-infected almond trees, increasing the frequency of antimicrobial peptide treatments led to greater reductions in bacterial populations and disease symptoms, whereas a single treatment was not curative (Moll et al., 2022). Likewise, field trials in olive have shown that repeated trunk injections of a Zn/Cu-based biocomplex (Dentamet^®^) were needed to suppress Xf populations over time, yet even then the bacterium could persist at low levels (Portaccio et al., 2025). In our study, the most pronounced differences between treated and untreated plants were observed for Xff: MCLE-treated plants infected with Xff maintained bacterial loads around 10^1–^10^2^ CFU, compared to ~10^5^ CFU in untreated controls. This outcome may reflect differential susceptibility among Xf subspecies or variation in their colonization behavior and ecology. Xff might be intrinsically more sensitive to certain bioactive compounds in MCLE due to differences in its cell envelope or metabolism (Vanhove et al., 2019). Alternatively, Xff may predominantly colonize more accessible xylem vessels in *N. benthamiana*, allowing the extract to penetrate and exert its effects, whereas Xfp and Xfm could form more extensive or deeper-seated biofilms that confer greater protection (De Souza et al., 2020). Further comparative studies of Xf subspecies biology will be useful to elucidate why their responses to treatments like MCLE differ.

Several plant models have been used historically to study Xf pathogenesis. After its first isolation in periwinkle (*Catharanthus roseus*) (Ueno et al., 1998), that species was developed as a model host (Monteiro et al., 2001), although in periwinkle typical leaf scorch symptoms can take months to develop. Comparable timelines were observed when *Medicago sativa* (alfalfa) was inoculated with Xfp strain De Donno (Abou Kubaa et al., 2019), although systemic movement in alfalfa was limited. More recently, *Arabidopsis thaliana* has been shown to be a useful experimental host: Pereira et al. (2019) demonstrated that *A. thaliana* can be systemically colonized by Xf (subsp. *pauca* and *fastidiosa*), with the bacteria spreading against xylem flow and inducing accumulation of anthocyanin pigments in infected leaves (Pereira et al., 2019). The ability to infect *Arabidopsis* (a genetically tractable model) provides opportunities to investigate plant innate immunity and genetic resistance factors against Xf. Our choice of *N. benthamiana* was motivated by its prior use as a rapid and reliable model for Xf: it supports high bacterial titers and expresses clear, quantifiable leaf scorch symptoms in a matter of weeks (Francis et al., 2008), as opposed to months or years in woody hosts. This makes *N. benthamiana* particularly amenable to testing therapeutics such as MCLE under controlled conditions. Indeed, *Nicotiana* species (including both *N. tabacum* and *N. benthamiana*) have been adopted in several recent Xf studies to evaluate control strategies (e.g. biocontrol endophytes or antimicrobial peptides) before transitioning to perennial hosts (Baró et al., 2022).

Because Xf is a xylem-limited pathogen, foliar spraying of treatments is largely ineffective – the active compounds must be delivered into the plant’s vascular system to reach the bacteria. For systemic plant pathogens, trunk injection has been applied successfully in other pathosystems, such as fire blight in apple (caused by *Erwinia amylovora*) and huanglongbing in citrus (*Candidatus Liberibacter* spp.) (Aćimović et al., 2014; Puttamuk et al., 2014). Analogously, trunk injection or endotherapy is being explored as a means to deliver anti-Xylella substances into infected or high-value host plants (e.g. olive trees). In this context, nanotechnology-based delivery systems may greatly enhance the efficacy of MCLE or similar plant extracts in field applications. Advanced formulations such as nanoparticles, nanoemulsions, or encapsulation in biocompatible polymers could improve the extract’s stability, bioavailability, and targeted distribution within xylem vessels. Nevertheless, such nanodelivery methods may not be directly applicable for crude leaf extracts, which comprise heterogeneous mixtures of various organic components with different physicochemical properties. The identification of specific active components of the extract is therefore required before nanoformulation.

A 2025 review by Portaccio et al. identified N-acetylcysteine and zinc/copper formulations as among the most effective treatments for Xf that are potentially field-deployable, while also noting that antimicrobial peptides and nanomaterials have shown high efficacy *in vitro* but require further field validation. We foresee that combining MCLE with nanocarriers or integrating it into biodegradable polymers for trunk injection could enhance its uptake and persistence inside plants. Not only would this ensure that the bioactive compounds reach the remote capillaries of the xylem where Xf resides, but it could also provide controlled release over time, maintaining effective concentrations. Equally important, such approaches would minimize the environmental impact compared to foliar sprays, since the treatment is confined within the plant. Overall, nanocarrier-enabled delivery of natural antimicrobials represents a cutting-edge, sustainable strategy for managing Xf and other vascular diseases. This approach is in harmony with the European Union’s push for innovative and eco-friendly plant protection solutions. Our findings with MCLE contribute to this emerging framework of sustainable Xf control.

## 5 Conclusion

In conclusion, we propose that MCLE represents a promising, reliable, and effective remedy against severe infections caused by *Xylella fastidiosa* subspecies *pauca* (Xfp), *multiplex* (Xfm), and *fastidiosa* (Xff). Plant-derived extracts are increasingly recognized for their compatibility with environmentally sustainable agricultural practices. They offer a viable alternative or complementary strategy to conventional agrochemicals in integrated disease management programs. In this study, the use of *Nicotiana benthamiana* as a model host provided a practical initial assessment; however, it may not fully capture the disease dynamics in olive or other natural hosts. Although MCLE exhibited significant antibacterial activity, the specific bioactive constituents responsible for this effect have yet to be identified. Additionally, the short-term, single-treatment design of the *in planta* trials limits the extrapolation of these findings to field conditions, underscoring the need for extended, multi-application trials under realistic agricultural scenarios. Further investigations should focus on assessing the stability and systemic movement of MCLE in *N. benthamiana*, identifying its active components, and evaluating its potential efficacy in diverse Xf-host species. Notably, assessing the effectiveness of MCLE in controlling *X. fastidiosa* in olive trees will require long-term experimentation, given that infected plants may remain asymptomatic for prolonged periods. Despite these limitations, our findings provide a foundation for future research aimed at developing sustainable, plant-based strategies for the management of *X. fastidiosa*.

## Data Availability

The raw data supporting the conclusions of this article will be made available by the authors, without undue reservation.
